# Multiscale analysis of mechanical stress in muscle under static and dynamic loading

**DOI:** 10.52601/bpr.2025.250019

**Published:** 2025-12-31

**Authors:** Xue Ke, Yansong Lu, Linjing Peng, Lu Yu, Yifei Yao

**Affiliations:** 1 School of Biomedical Engineering, Shanghai Jiao Tong University, Shanghai 200030, China; 2 College of Biomedical Engineering and Instrument Science, Zhejiang University, Hangzhou 310027, China

**Keywords:** Cyclic force stimulation, Multiscale muscle hyper-viscoelastic models, Finite element analysis, Stress relaxation, Deep tissue injury

## Abstract

Deep tissue injury (DTI) is a serious condition primarily triggered by prolonged mechanical loading rather than short-term excessive force. Cyclic force stimulation has been shown to enhance cellular protection; however, the biomechanical mechanisms underlying tissue and cellular responses to such stimulation remain poorly understood. This study investigates the biomechanical effects of cyclic force transmission from viscoelastic muscle tissue to muscle fibers using a multiscale finite element model with varying cyclic force parameters. Finite element analysis was used to examine the impact of different frequencies and amplitudes of force stimulation, considering the viscoelastic properties of tissues and cells. Results indicated that, on each one of the three model scales, the average maximal Von Mises stress during stress relaxation increased with cyclic force amplitude at a constant frequency. At a fixed amplitude, frequency variations did not influence the average maximal Von Mises stress in the macroscopic and mesoscopic models. However, in the microscopic model, higher frequencies resulted in lower average maximal Von Mises stress at low amplitudes and higher average maximal Von Mises stress at high amplitudes. Notably, a high-frequency, low-amplitude cyclic force mode reduced the average maximal minimum principal stress by 3.6% in the first four seconds and 5.8% in the last 16 seconds of the 20-second stress relaxation period. These findings suggested that such a cyclic force mode may mitigate or delay mechanical damage caused by prolonged mechanical loading, offering insights into potential strategies for preventing DTI.

## INTRODUCTION

Individuals who are bedridden, wheelchair-bound, or dependent on prostheses for extended periods due to illness or other conditions are at high risk of developing severe complications associated with prolonged immobility. Among these, pressure ulcers are a widespread and serious concern.

Pressure ulcers, also known as pressure injuries, are clinical conditions characterized by tissue rupture, ulceration, and necrosis caused by prolonged mechanical loading on the body’s tissues (Bouten *et al.* 2005). Their primary pathophysiological responses include local ischemia, reperfusion injury, impaired lymphatic drainage, and cellular deformation (Bouten *et al.* 2003). Pressure ulcers can manifest as either superficial or deep tissue injuries. Superficial ulcers primarily affect the upper layers of the skin, resulting from shear stress within the skin layers, whereas deep tissue injury (DTI) arises due to sustained pressure, particularly in tissues underlying bony prominences (Nola and Vistnes 1980).

Muscle tissue is significantly more susceptible to mechanical loading than skin tissue, with a lower compression injury threshold (Daniel *et al.* 1981). The duration for which muscle tissue can withstand mechanical loads depends on the intensity of the applied pressure (Yao *et al.* 2015), and its tolerance varies among individuals due to differences in physiological sensitivity (Coleman *et al.* 2014). While excessive short-term mechanical loads can cause rapid muscle cell necrosis (Breuls *et al.* 2003), deep tissue injuries are more commonly attributed to prolonged, localized mechanical loading over bony structures beneath the skin. The risk and severity of these injuries are influenced by the type, magnitude, and duration of epidermal loading (Mak *et al.* 2011).

Pressure ulcers are particularly prevalent among sedentary populations worldwide, leading to long-term physical pain, psychological distress, and even severe disability or mortality in elderly individuals. Additionally, the prolonged recovery time and intensive care required for pressure ulcer management impose a significant economic burden on healthcare systems. Epidemiological studies across different geographic regions highlight the increasing prevalence of pressure ulcers, underscoring the need for effective prevention strategies (Haalboom 2005; Brandeis *et al.* 1990; Tubaishat *et al.* 2018; Jiang *et al.* 2014; Wei *et al.* 2021).

Recent research suggests that cyclic force stimulation can enhance cellular protection, improve muscle resistance to mechanical damage, and reduce the risk of deep tissue injury formation. At the molecular level, cyclic mechanical stimulation modulates the expression of key proteins and enzymes. Prolonged cyclic vibration influences factors such as PGC-1α, catalase, Gpx-1, and SOD1, which maintain enzymatic antioxidant defense mechanisms and inhibit oxidative stress, thereby reducing compression-related muscle damage (Wong *et al.* 2017). In animal models, cyclic mechanical strain suppresses the expression of hypoxia-inducible factors (HIF-1), MMP2, and MMP9, mitigating hypoxia-induced muscle deterioration (Sari *et al.* 2015). Additionally, cyclic uniaxial tensile strain upregulates mechanical growth factor (MGF), promoting myoblast proliferation (Li *et al.* 2009). Under cyclic tensile stress, protein expression of α-actinin increases in short-term feedback, improving cell adhesion, adapting to mechanical stimuli, and reducing cell damage (Wang *et al.* 2010). Other studies indicate that cyclic stretching enhances nitric oxide production, regulates COX-2 expression, and facilitates myogenic cell proliferation (Soltow *et al.* 2010). Moreover, cyclic force stimulation influences β1D integrin expression, affecting muscle differentiation and survival (Zhang *et al.* 2007), while also promoting vascular smooth muscle proliferation by modulating microRNA-19b-3p and connective tissue growth factor (CTGF) expression (Wang *et al.* 2019).

At the cellular level, cyclic force stimulation affects both the structure and function of cells. Previous research has shown that it enhances cellular resistance to stress, accelerates membrane repair, reduces tensile strain, and promotes tissue regeneration (Yao and Mak 2016). Cyclic mechanical strain also stimulates myofibroblast proliferation, myotube maturation, and sarcomere assembly (Dugan *et al.* 2014), while potentially influencing myoblast elasticity and differentiation capacity (Takemoto *et al.* 2012). In tissue engineering, cyclic loading facilitates fibroblast proliferation and extracellular matrix deposition, allowing engineered tissues to better replicate the mechanical properties of natural tissues (Wang *et al.* 2004).

Finite element analysis (FEA) is a widely used computational method for simulating the biomechanical response of tissues and cells under different mechanical conditions. By solving partial differential equations numerically, FEA enables the investigation of complex mechanical interactions in biological systems. Previous studies have employed FEA to model cellular responses under static and cyclic loading. For instance, Wang et al. treated cells as linear elastic materials under static loading (Wang *et al.* 2015), while Bursa *et al*. modeled vascular smooth muscle cells using the homogeneous isotropic hyperelastic intrinsic material model parameters (Bursa *et al.* 2006). More advanced models incorporate viscoelastic properties to capture creep, relaxation, and hysteresis behaviors, which are critical for understanding cell deformation under cyclic force stimuli (Zhu *et al.* 2000). Peeters *et al*. measured the viscoelastic properties of individual C2C12 myoblasts under cyclic compression (Peeters *et al.* 2005), while Wang *et al*. used a finite element viscoelastic model to analyze osteoblast responses to vibrational stimuli, highlighting the importance of viscoelasticity in biomechanical simulations (Wang and Xian 2016).

Multiscale modeling provides further insights into biomechanical processes by integrating analyses across different length scales. Prior studies on ligament and tendon mechanics have demonstrated how viscoelastic responses vary across nanoscale protofibrils, microscale fibers, mesoscale bundles, and whole-tissue structures (Reese and Weiss 2015). Similarly, Cen *et al*. examined age-related differences in femoral mechanics by investigating the hierarchical transfer of mechanical forces from the whole bone down to osteocytes (Cen *et al.* 2021). These multiscale approaches enable a more comprehensive understanding of stress distribution and mechanical adaptation within biological tissues.

By leveraging multiscale modeling, complex biomechanical problems can be decomposed into distinct components, allowing for more precise analysis of mechanical interactions at different structural levels. Compared to single-scale models, multiscale simulations provide deeper insights into continuous mechanical transfer processes, offering potential applications in disease prevention and treatment from the organ to the cellular and molecular levels.

Given the viscoelastic nature of tissues and cells, the mechanical response to cyclic force stimuli remains an underexplored area of research. This study aimed to investigate the biomechanical effects of cyclic force transmission from viscoelastic muscle tissue to muscle fibers using a multiscale finite element model with varying cyclic force parameters. By integrating principles of mechanical vibration and multiscale biomechanics, this research sought to provide new insights into tissue injury prevention and rehabilitation, while establishing standardized modeling and simulation paradigms for future studies on tissue biomechanics.

## RESULTS

### Model validation

#### Validation of the consistency of micro-meso-mechanical parameters

The microscopic model was fixed at one end surface, and a strain-displacement load of 5% was applied to the opposite surface. This setup allowed for the generation of stress-strain curves up to 5% strain for the extracellular matrix-muscle fiber composite in the microscopic model, as well as the time-dependent viscoelastic stress relaxation curves.

**Figure 1:**
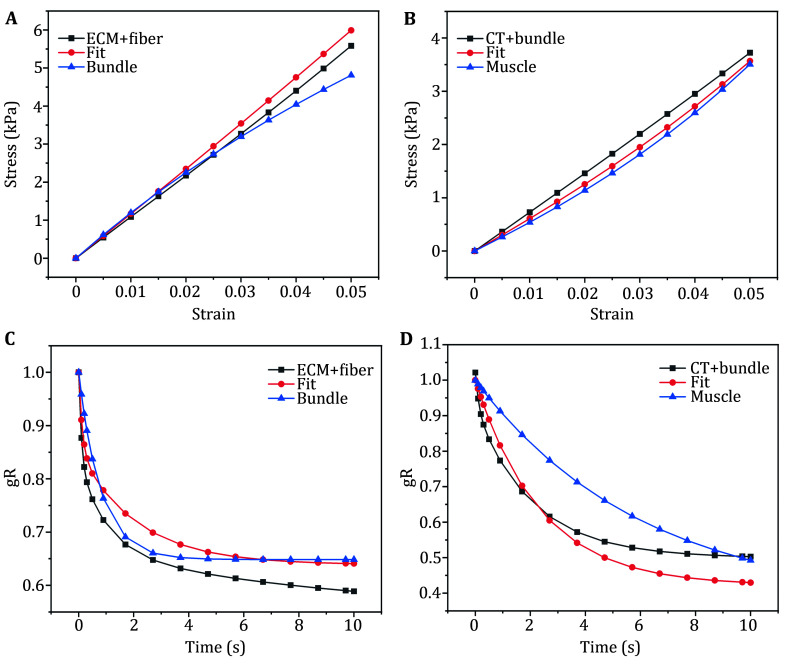
**A** Stress-strain curve of muscle bundle, and fitted as well as original ECM-fiber complex hyperelasticity consistency validation. ECM: extracellular matrix. **B** Stress-strain curve of muscle, and fitted as well as original CT-bundle complex hyperelasticity consistency validation. CT: connective tissue. **C** Stress relaxation curves for consistency validation of muscle bundle, and fitted as well as original ECM-fiber complex viscoelasticity. ECM: extracellular matrix. **D** Stress relaxation curves for validation of muscle, and fitted as well as original CT-bundle complex viscoelastic consistency. CT: connective tissue

The hyperelastic parameters of the muscle bundles in the mesoscopic model were fitted using the stress-strain relationship of the composite, and the results were compared with the hyperelastic parameters of muscle bundles, as shown in Fig. 1A. Error calculations were performed using 10 strain points, which showed an error of 9.82% between the fitted hyperelastic stress-strain curve of the muscle bundles and that of the extracellular matrix-muscle fiber composite. This indicated that the fitted hyperelastic parameters provided a good approximation of the muscle bundle parameters.

In addition, the time-dependent stress relaxation curves within 10 seconds for the composite were obtained to fit the viscoelastic parameters of the muscle bundles in the mesoscopic model. These curves were compared with the viscoelastic parameters of muscle bundles from the literature, as shown in Fig. 1C. Error calculations were made at 15-time points, and the fitted viscoelastic stress relaxation curves showed an error of 3.02% compared to that of the muscle bundle. This confirmed that the fitted viscoelastic parameters were a very good fit for the muscle bundle material property parameters.

The fitted hyperelastic and viscoelastic parameters of the muscle bundles were then used as the mechanical properties for the muscle bundles in all subsequent simulations and for validating the consistency of the parameters between the mesoscopic and macroscopic models.

#### Validation of meso-macro mechanical parameter consistency

The model was fixed at one end surface, and a strain-displacement load of 5% was applied at the opposite surface end. This setup generated stress-strain curves up to 5% strain and time-dependent viscoelastic stress-relaxation curves for the mesoscopic muscle-bundle-connective-tissue complex.

The hyperelastic parameters of the muscle in the macroscopic model were fitted using the stress-strain relationship of the complex and compared with the muscle hyperelastic parameters, as shown in Fig. 1B. Error calculations were performed using 10 strain points, revealing an error of 6.75% between the fitted muscle hyperelastic stress-strain curve and the muscle hyperelastic parameters. This indicated that the fitted muscle hyperelastic parameters provided a good approximation of the muscle bundle hyperelasticity parameters.

Furthermore, the time-dependent stress relaxation curves of the composite within 10 seconds were obtained to fit the viscoelastic parameters of the muscle bundles in the mesoscopic model. These curves were compared with the viscoelastic parameters of muscle bundles, as shown in Fig. 1D. Error calculations were performed using 15-time points, showing an error of 13.24% between the fitted viscoelastic stress-relaxation curves of the muscle bundles and that of the viscoelastic muscle. This suggested that the fitted muscle bundle viscoelastic parameters were a reasonable fit.

The fitted hyperelastic and viscoelastic parameters for the muscle were then used as the mechanical properties in all subsequent simulations.

#### Validation of the validity of the macroscopic pseudo-3D model

This study utilized a pseudo-3D macroscopic buttock model for subsequent simulations. To ensure the validity of this model in reflecting the real human buttock scenario, validation was necessary. In our laboratory, we measured the cushion surface contact stresses in adult males during buttock-cushion contact using the BPMS pressure sensor (Jia *et al.* 2023). Additionally, we employed the inverse finite element analysis method to assess the shear stresses in muscle and fat based on the actual measured stress relationships.

**Figure 2:**
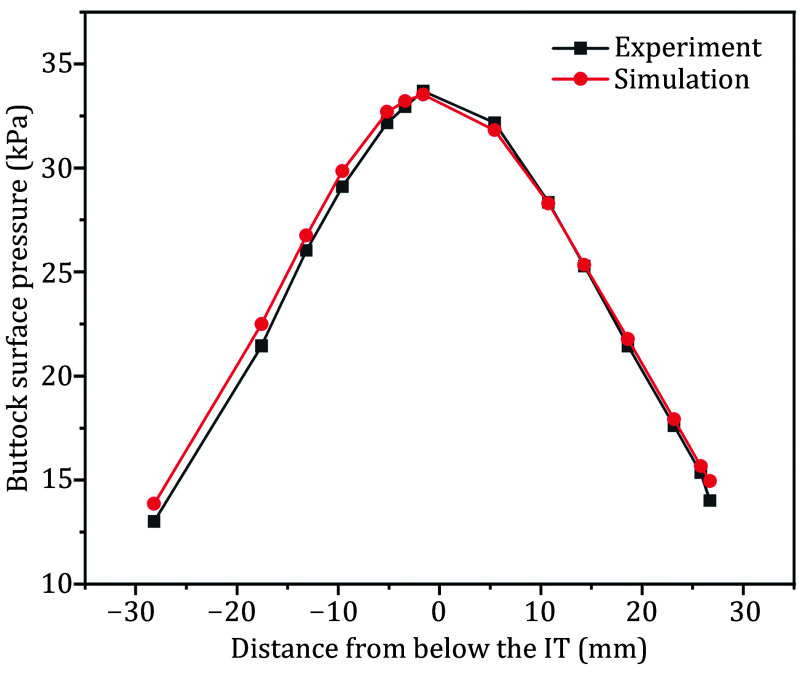
Comparison of simulation and experimental buttock surface pressure data. IT: ischial tuberosity

For validation, the contact stress on the buttock-cushion contact surface was obtained by pressing down the sciatic tuberosity in the macroscopic model by 14.1 mm, as in the experimental setup. These results were then compared with experimental data from the literature, as shown in Fig. 2. In this figure, the horizontal axis represents the bottom of the sciatic tuberosity as the initial zero point, and the vertical axis shows the contact stress on the surface of the buttock. The error between the simulated and experimental data was calculated to be 2.28%, suggesting that the pseudo-3D gluteal model can effectively reflect the real situation of the buttock-cushion contact.

### Static analyses

#### Macro model

A displacement of 14.1 mm was applied to the sciatic tuberosity to simulate the effect of the upper body’s weight in a seated position on the sciatic tuberosity and various tissue regions, such as muscle and fat. Due to the viscoelastic property of the model material, which introduces time-dependent stress decay, the first analysis step was designed so that the 14.1 mm displacement was applied to the sciatic node within the first 0.1 s. In the second analysis step, which lasted 20 s, the displacement of the sciatic node was held constant, allowing the model to reach a stress relaxation state.

**Figure 3:**
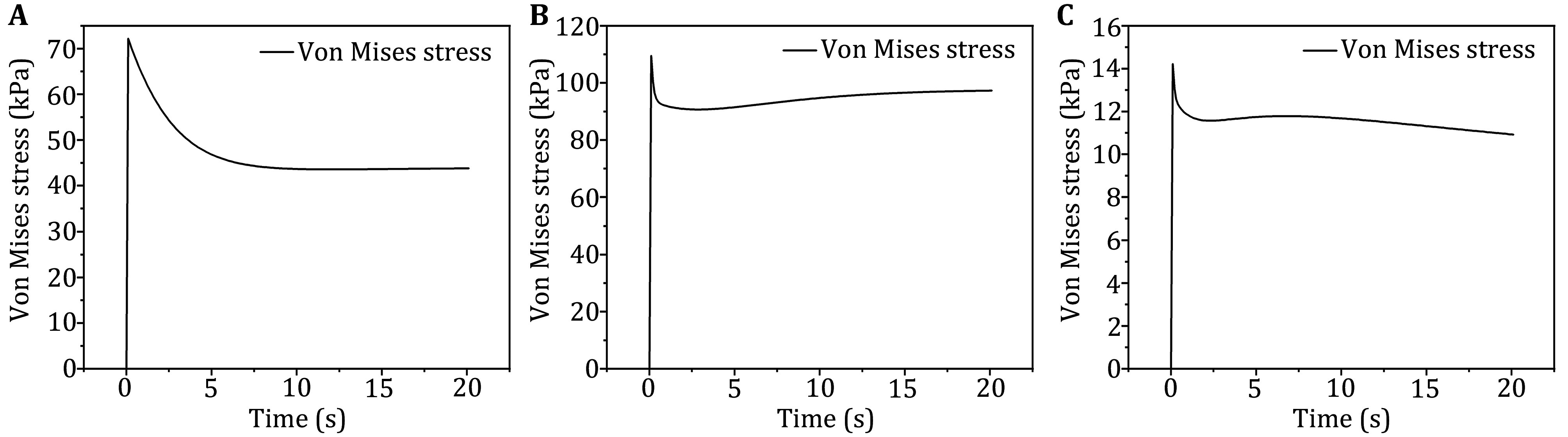
Stress-relaxation curves for nodes with maximal Von Mises stress within macroscopic muscle (**A**), mesoscopic muscle bundle (**B**), and microscopic muscle fiber (**C**)

To identify the region experiencing the maximal Von Mises stress, the muscle region below the sciatic node was searched, excluding any nodes where boundary conditions might cause anomalies. The stress-time curves for the nodes within this region were then plotted, as shown in Fig. 3A. From 0 to 0.1 s, under the applied displacement loading, the Von Mises stress increased rapidly from 0 to 72 kPa, dominated by the hyperelastic behavior of the material. Between 0.1 and 20.1 s, the Von Mises stress gradually decayed from 72 kPa to approximately 44 kPa, due to the time-dependent viscoelastic relaxation and the small displacement of the muscle tissues during this phase. There was a slight increase in Von Mises stress during the relaxation phase due to the small displacements occurring within the muscle tissues.

The time constant for muscle viscoelastic decay was found to be 2.4 s. To better analyze the stress relaxation process, the 20 s period was divided into two stages: the first 4 s representing the rapid stress decay phase, and the last 16 s corresponding to the slow stress change phase. The average maximal Von Mises stress during these phases was calculated separately. The average maximal stress for the first 4 s was 57.4 kPa, and for the last 16 s, it was 44.2 kPa. These values were used to assess the damage within the statically loaded muscle. Additionally, the time-dependent relative displacements transmitted from the macroscopic scale to the mesoscopic scale were also calculated.

#### Mesoscopic model

To align with the analysis step and displacement settings of the macroscopic model, a downward displacement of 2 mm was applied to the upper surface of the mesoscopic model from 0 to 0.1 s. From 0.1 to 20.1 s, a continuous displacement loading was input into the magnitude table of Abaqus, based on the relative displacement in the y-axis direction of the region with the maximal Von Mises stress obtained from the macroscopic model, as a function of time.

The region of the muscle bundle with the maximal Von Mises stress was identified, excluding any nodes where boundary condition anomalies might occur, and the stress-relaxation curves for the nodes in that region were plotted, as shown in Fig. 3B.

From 0 to 0.1 s, the Von Mises stress rapidly increased from 0 to 109.4 kPa due to the dominant hyperelastic effect. From 0.1 to 2.8 s, the Von Mises stress gradually decayed from 109.4 kPa to approximately 90.7 kPa, as a result of the time-dependent viscoelastic relaxation. However, due to the small displacement during the stress relaxation phase of the macroscopic model, the Von Mises stress in the mesoscopic model continued to increase after 2.8 s, under the combined effects of small compression and time-dependent viscoelastic decay. By 20.1 s, the stress increased to 97.2 kPa. The stress change in the muscle bundles followed the same two-phase process observed in the macroscopic model. The average maximal Von Mises stresses in the two phases were calculated separately. The average maximal Von Mises stress during the first 4 s was 91.6 kPa, and the average maximal Von Mises stress during the last 16 s was 95.1 kPa. These values were used to assess internal damage within the statically loaded muscle bundles. Additionally, the time-dependent relative displacement transferred from the mesoscopic scale to the microscopic scale was also calculated.

#### Microscopic model

To align with the analysis step and displacement settings of the macroscopic and microscopic models, a compressive displacement loading of 0.34 mm was applied during the first analysis step (0 to 0.1 s). In the second analysis step (0.1 to 20.1 s), relative displacements, obtained from the mesoscopic model, were used to simulate compression ranging from 1.34 to 1.39 mm over time.

The region of the muscle fibers experiencing the maximal Von Mises stress was identified, excluding any nodes where boundary condition anomalies might occur. Stress-time curves were plotted for the nodes within this region, as shown in Fig. 3C.

From 0 to 0.1 s, the Von Mises stress rapidly increased from 0 to 14.2 kPa, driven by the dominant hyperelastic effect. From 0.1 to 20.1 s, the Von Mises stress showed an initial increase followed by a decrease and eventual stabilization, decaying to 10.9 kPa. This behavior is influenced by the time-dependent viscoelastic decay and the small displacement effect from the macroscopic model's stress relaxation phase on the microscopic model. The stress-time process in the muscle fibers mirrored the two stages observed in the macroscopic model. The average maximal Von Mises stresses for the two phases were calculated separately, with an average of 11.8 kPa for the first 4 s and 11.5 kPa for the last 16 s. These values were used for the assessment of damage in the statically loaded muscle fibers.

### Dynamic analyses

#### Macroscopic model

**Figure 4:**
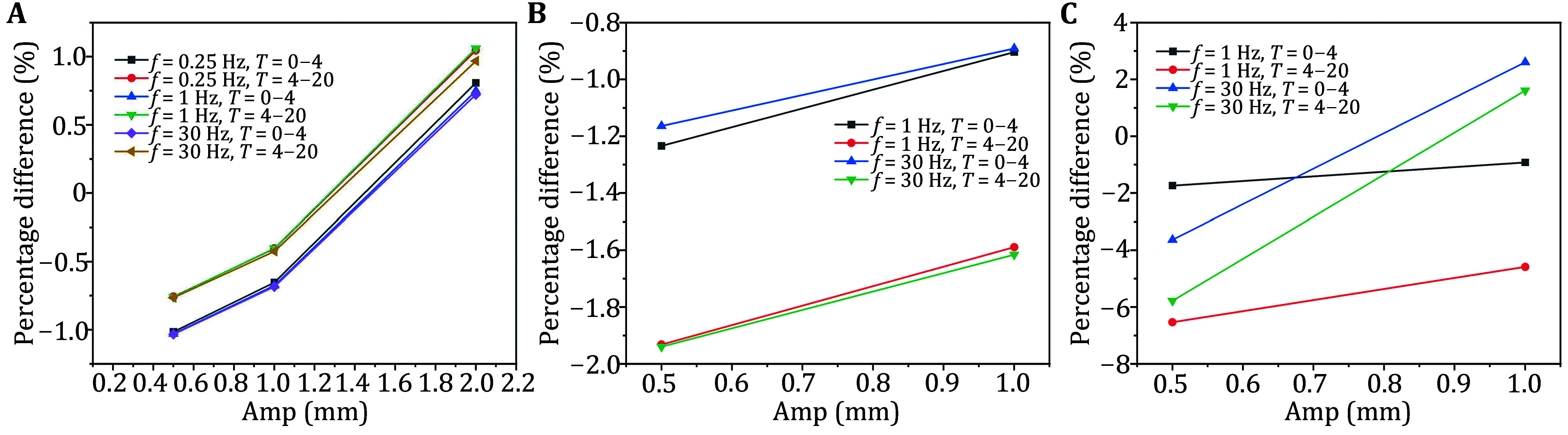
Average maximal Von Mises stress variation under different amplitudes (same frequency) at macroscopic (**A**), mesoscopic (**B**), and microscopic scales (**C**)

As shown in Fig. 4A for the same vibration frequencies ranging from 0.25 to 30 Hz, the average maximal Von Mises stress in the region increased with the vibration amplitude. Even at a vibration amplitude of 2 mm, the average maximal Von Mises stress for cyclic displacement loading exceeds the corresponding value for static loading. This suggested that the muscle tissue became more susceptible to reaching the damage stress threshold with increased vibration amplitude.

At a vibration frequency of 0.25 Hz, when the vibration amplitude changes from 0.5 to 1 mm, the stress reduction difference between the two-time phases was 0.362% and 0.354%, respectively. When the vibration amplitude changes from 1 to 2 mm, the stress reduction difference between the two-time phases was 1.461% and 1.449%, respectively. For a vibration frequency of 1 Hz, when the vibration amplitude changed from 0.5 to 1 mm, the stress reduction difference was 0.354% and 0.357%, respectively. When the vibration amplitude changed from 1 to 2 mm, the stress reduction difference was 1.424% and 1.463%, respectively. At 30 Hz, when the vibration amplitude changed from 0.5 to 1 mm, the stress reduction difference between the two-time phases was 0.349% and 0.340%. When the vibration amplitude changed from 1 to 2 mm, the difference was 1.407% and 1.394%, respectively.

From these results, as the vibration amplitude increased at different frequencies, the trend in the average maximal Von Mises stress over the two-time phases remained almost the same. In other words, the overall trend of stress change during the relaxation process was consistent, and with an increase in amplitude, the average stress changed more quickly.

**Figure 5:**
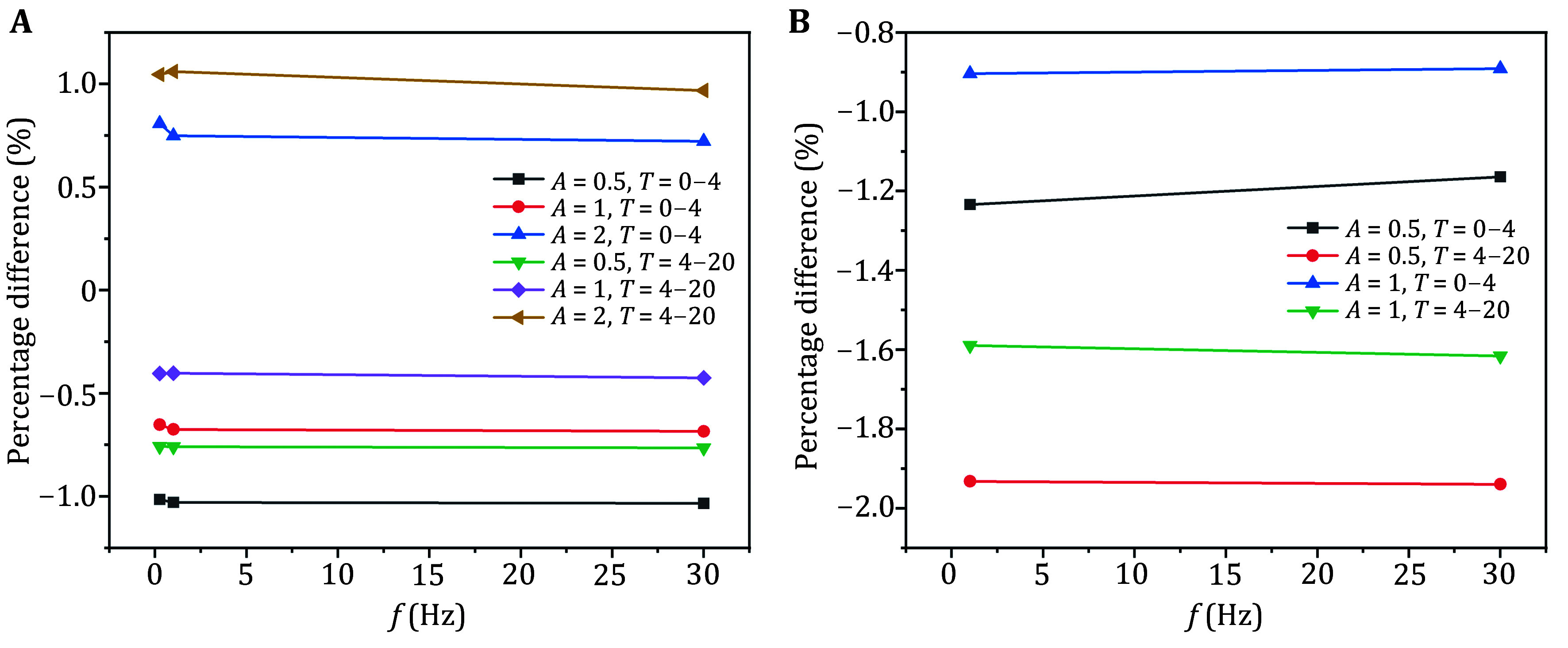
Average maximal Von Mises stress variation under different frequencies (same amplitude) for macroscopic (**A**) and mesoscopic models (**B**)

As shown in Fig. 5A, under the same vibration amplitude, the average maximal Von Mises stress changed during both time phases – averaged above and below different vibration frequencies – and remained almost identical. This indicated that varying the vibration frequency has little effect on the stress intensity in the stress relaxation curve at the macroscopic scale.

#### Mesoscopic model

As shown in Fig. 4B, within the frequency range of 1 to 30 Hz, for each time stage at the same frequency, a larger vibration amplitude results in a smaller reduction effect of cyclic displacement loading on the average maximum Von Mises stress. Similar to the conclusion observed at the macroscopic scale, at the mesoscopic scale, the stress changed during the two-time phases – when different vibration amplitudes were applied – were comparable to those under static loading. This indicated that the stress relaxation across the phases was similar to that observed under static loading and was almost independent of the vibration frequency.

As shown in Fig. 5B, under the same vibration amplitude, the average maximal Von Mises stress changed during the two phases – averaged across different vibration frequencies – were nearly identical. This suggested that changes in vibration frequency had little effect on the stress intensity of the stress relaxation curves at the mesoscopic scale.

#### Microscopic model

As shown in Fig. 4C, within the frequency range of 1 to 30 Hz, a larger vibration amplitude resulted in a larger average maximal Von Mises stress for each time stage at the same frequency. Additionally, at the same frequency, the average maximal Von Mises stresses at different vibration amplitudes for both time phases followed the same trend as in static loading. However, unlike the macroscopic and mesoscopic scales, the magnitude of the change varied significantly across different frequencies. It can be observed that compared to 1 Hz, 30 Hz results in a more significant change in the average maximal Von Mises stress with respect to static loading when the amplitude was varied. For instance, at an amplitude of 0.5 mm, the simulation results at 30 Hz showed a greater reduction in average maximal Von Mises stress compared to static loading than at 1 Hz. However, when the amplitude was increased to 1 mm, the average maximal Von Mises stress at 30 Hz was slightly higher than that under static loading. These findings suggested that high-frequency, low-amplitude vibration may be more effective in preventing damage and reducing the mean maximum Von Mises stress, thereby helping to prevent or delay muscle fiber damage.

Additionally, when considering the average maximal minimum principal stress, the use of high-frequency, low-amplitude vibration resulted in a reduction of 3.6% in the first 4 s and 5.8% in the last 16 s during the 20-s stress relaxation process. This suggested that high-frequency, low-amplitude macro-vibration could potentially prevent or delay mechanical damage caused by short-term excessive mechanical loading by reducing the average maximal minimum principal stress in the first 4 s of stress relaxation. It could also help avoid or delay mechanical damage due to long-term mechanical loading by decreasing the average maximal minimum principal stress in the last 16 s. These results indicated that muscle fibers might be more susceptible to mechanical damage from long-term loading when the average minimum principal stress was reduced during the early phase of stress relaxation, particularly within the first 4 s.

## DISCUSSION

This study investigated the impact of different vibration frequencies and amplitudes on the average maximal Von Mises stresses under cyclic displacement loading. High-frequency vibration at the microscale has a more significant effect on the average maximal Von Mises stress compared to low-frequency vibration. For the macroscale and mesoscale, the objects of the study are muscles and muscle bundles, respectively, with shear moduli of approximately 18 kPa for muscles and 38 kPa for muscle bundles. At the microscale, muscle fibers are studied, with a shear modulus of about 4 kPa. A lower shear modulus indicates a higher sensitivity to deformations. Furthermore, the viscoelastic decay of muscle fibers at the micro-scale is faster than that of muscles and muscle bundles at the macro- and mesoscopic scales. During rapid viscoelastic decay in stress, high-frequency vibration has a more pronounced effect on the average maximal Von Mises stress for muscle fibers. High-frequency vibration also has a stronger influence on tissues with low shear modulus when the viscoelastic time constant is small. At the macroscopic and mesoscopic scales, however, the effects of high-frequency vibration and low-frequency vibration on the average maximal Von Mises stresses of muscles and muscle bundles are less pronounced due to their higher shear modulus and relatively slower viscoelastic time decay in stress.

Regarding vibration amplitude, at the macro-scale, and for the micro- and mesoscopic scales, there are instances where the average maximal Von Mises stress at low amplitude is lower than that at high amplitude. In fact, at high amplitudes, the average maximal Von Mises stress at certain scales may exceed the corresponding value under static loading. Therefore, it is important to account for the possibility that high amplitudes may result in larger average maximal Von Mises stresses compared to static loading, leading to more risk of tissue damage.

For practical applications, the selection of vibration frequency and amplitude should take both biological and mechanical effects into account. For instance, Ichioka *et al*. found that high-frequency vibration may help increase blood microcirculation (Ichioka *et al.* 2011). Additionally, Wong *et al*. discovered that high-frequency, low-intensity vibration could enhance antioxidant defense in mouse muscles, reducing compression-induced oxidative damage (Wong *et al.* 2017). Low-frequency vibration has also been associated with cellular and molecular biological effects. For example, Dugan *et al*. suggested that low-frequency vibration could promote cell proliferation by up-regulating mechano-growth factors in myofibroblasts (Dugan *et al.* 2014), while Wang *et al*. suggested that low-frequency vibration could regulate myofibroblast adhesion by controlling the expression of α-actinin (Wang *et al.* 2010).

Yao *et al*. measured the compression damage threshold of C2C12 mouse myoblasts (Yao *et al.* 2015). The damage threshold follows a sigmoidal curve over time, with excessive mechanical loading causing direct myoblast damage in the short term. Over the long term, smaller mechanical loads can also lead to myoblast damage. In this study, static loading and cyclic displacement loading were analyzed for the region of maximal minimum principal stress within muscle fibers. It is worth noting that the minimum principal stress, as the largest compressive principal stress component, directly quantifies the directional compressive loading. While Von Mises stress evaluates overall stress intensity regardless of direction, the minimum principal stress specifically captures the maximum stress acting along the principal compressive axis, making it a more precise metric for assessing compression-dominated tissue damage mechanisms such as deep tissue injury. This distinction ensures that the analysis aligns with the physiological relevance of compressive forces in pathological processes. Under static loading, the average maximal compressive stress of muscle fibers was 11.1 kPa in the first time phase (0 to 4 s) and 7.6 kPa in the second time phase (4 to 20 s). Under high-frequency, low-amplitude vibratory loading, the average maximal compressive stress decreased by 3.6% in the first time phase (0 to 4 s) and 5.8% in the second time phase (4 to 20 s). Comparing these results with the damage threshold curves, if the damage thresholds of muscle fibers are similar to those of myoblasts, high-frequency, low-amplitude macro-vibration may reduce the compressive stress in muscle fibers during the first time phase, thereby helping to avoid or delay mechanical damage from short-term excessive loading. It could also reduce the compressive stress in the second time phase, preventing or delaying mechanical damage due to long-term loading.

It should be noted that muscle fibers are tougher than individual myoblasts, and this study preferred the material parameters of aged muscle, which exhibit greater stiffness than non-aged muscle. The damage thresholds of muscle fibers in skeletal muscle under short-term excessive and long-term sustained loading remain unclear, and further research is needed to measure these thresholds under different aging conditions. This will help verify the effect of high-frequency, low-intensity vibration on reducing the compressive stress in muscle fibers, as presented in this study.

This study has several limitations that should be addressed in future investigations. First, the material properties for the macroscopic, mesoscopic, and microscopic models were sourced from various studies, some based on animal experiments. The heterogeneity of these sources may introduce errors in the finite element simulations. Future work should include direct experimental measurements across all scales to minimize variability. Second, the geometric models were based on assumptions that may not fully represent individual anatomical differences. Variations in anatomy and seating posture can alter hemipelvic geometry, impacting the dimensions and morphology of mesoscopic and microscopic models, although the pseudo-3D macroscopic model was validated against measured surface pressures. Finally, this study relied solely on idealized finite element simulations without experimental validation on the microscopic scale. However, *in vivo* force measurement on the cellular level is almost unavailable. Future research should develop innovative approaches to measure the cellular force *in vivo* instead of the current only approach through multiscale biomechanical simulation to calculate the force on cellular level.

## CONCLUSION

This study developed models at macroscopic, mesoscopic, and microscopic scales to examine mechanical processes under static and cyclic dynamic compressive loading on muscle. Results showed that vibration frequency has minimal impact on Von Mises stress across scales, while vibration amplitude significantly increased stress, particularly at 2 mm amplitude. At the microscopic scale, high-frequency vibrations cause greater stress variation due to the smaller shear modulus and faster viscoelastic decay in the stress of muscle fibers. High-frequency, low-amplitude vibrations are effective in reducing compressive stress, potentially preventing or delaying muscle damage from both short-term excessive and long-term sustained loading.

## MATERIALS AND METHODS

### Macro-scale pseudo-3D model of the gluteal region

#### Geometric modeling

A study analyzed strain and stress distribution in deep muscle and adipose tissue of the buttocks during sitting (Linder-Ganz *et al.* 2007). Using MRI coronal plane images of the subjects’ buttocks, they identified three distinct tissue regions: muscle, adipose tissue, and the sciatic node. Comparing non-weight-bearing and weight-bearing sitting postures, they found that the muscle and fat regions beneath the sciatic node experienced the highest mechanical loads and strains. This insight provided a reference for defining the region of interest in the macroscopic model.

**Figure 6:**
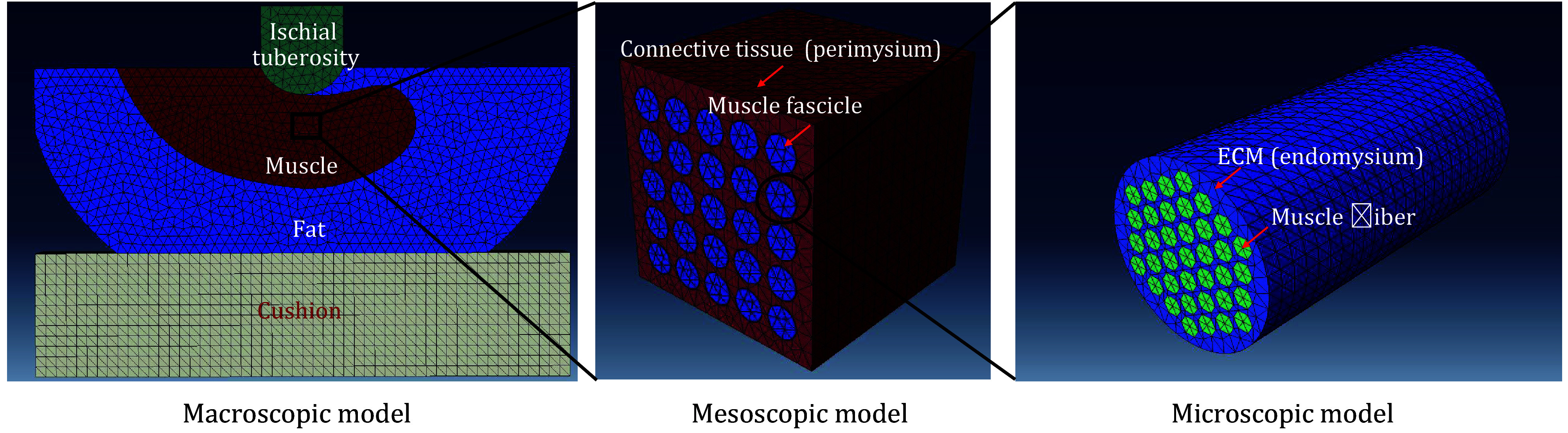
Schematic diagram of macroscopic, mesoscopic and microscopic model components

In this study, a macroscopic pseudo-3D model of the gluteal region was developed based on the MRI coronal sections (Linder-Ganz *et al.* 2007). The MRI images were segmented into subregions, and different tissue areas were outlined using SolidWorks. The model was then extruded to create a pseudo-3D representation. Given the symmetrical nature of the sciatic tuberosity region, only half of the lateral region was modeled, with a symmetric boundary condition applied during analysis. The final model, shown in Fig. 6, consists of four distinct parts: sciatic tuberosity, muscle, fat, and cushion.

The overall macroscopic model measures 128 mm in length, 100 mm in width, and 4 mm in thickness. The muscle beneath the sciatic tuberosity has a thickness of 22 mm, while the adipose layer measures 14 mm.

#### Mechanical parameters

To accurately describe the mechanical behavior of muscle and adipose tissues, both hyperelasticity and viscoelasticity must be considered. In this study, the shear modulus of adipose tissue was obtained from literature sources, assuming incompressibility. Using this information, the Neo-Hookean hyperelastic parameters for adipose tissue were derived. Additionally, the fourth-order Prony Series viscoelastic parameters were adopted from Then *et al*. (2012).

For muscle tissue, the Neo-Hookean hyperelastic and first-order Prony Series viscoelastic parameters were determined by fitting stress-strain and stress relaxation curves. These curves were validated against literature data and used to refine the mesoscopic model's hyperelastic and viscoelastic properties.

Tables 1 and 2 presented the elastic and viscoelastic mechanical parameters for different macroscopic tissue regions used in this study.

**Table 1:** Mechanical parameters in different regions of the macroscopic model

Region	Type	Constitutive model	ABAQUS parameter
Bone	Linear elastic	Linear elastic	$ E=20\;\mathrm{G}\mathrm{P}\mathrm{a};v=0.3 $
Cushion	Linear elastic	Linear elastic	$ E=3\;\mathrm{M}\mathrm{P}\mathrm{a};v=0.3 $
Muscle	Hyperelastic	Neo Hookean	$ C10=9.56\;\mathrm{k}\mathrm{P}\mathrm{a};D1=2.11 $
Fat	Hyperelastic	Neo Hookean	$ C10=16\;\mathrm{k}\mathrm{P}\mathrm{a};D1=1.26 $

**Table 2:** Viscoelastic mechanical parameters in different regions of the macroscopic model

Region	Type	Constitutive model	ABAQUS parameter (Then *et al.* 2012)
Muscle	Viscoelastic	Prony series	$ g1=5.867e-1;k1=0;\tau 1=2.3356 $
Fat	Viscoelastic	Prony series	$ g1=1.33e-2;k1=1.32e-2;\tau 1=2 $ $ g2=3.64e-3;k2=3.25e-4;\tau 2=4e+1 $ $ g3=3.85e-4;k3=5.6e-4;\tau 3=8e+1 $ $ g4=1.6e-2;k4=1.89e-5;\tau 4=2e+2 $

#### Mesh

A sensitivity analysis was conducted on the mesh of the macroscopic model. Given that the normal *in vivo* displacement of the sciatic tuberosity ranges from 10 to 15 mm (Xiao *et al.* 2014), a downward displacement loading of 14.1 mm was applied to the sciatic tuberosity to determine the total force exerted on the upper surface of the cushion, thereby validating the mesh sensitivity of the buttock model. Initially, the model consisted of 2429 elements for the sciatic node, 21,437 for the cushion, 16,521 for muscle, and 9567 for fat, resulting in a total force of 6.532 N on the upper surface of the cushion. When refining the mesh to 4975 cells for the sciatic node, 21,437 for the cushion, 38,872 for muscle, and 26,767 for fat, the total force was 6.529 N, yielding an error of just 0.02%. Across different mesh configurations, the error remained below 1%, confirming that the chosen mesh resolutions for the sciatic node, cushion, muscle, and fat were appropriate for the macroscopic model.

### Mesoscopic-scale muscle bundles and muscle bundle membranes complex

#### Geometric modeling

Based on the geometric characteristics of mesoscopic-scale muscle bundles and muscle bundle membranes (connective tissues), the muscle bundles were modeled as cylindrical structures wrapped by muscle bundle membranes. The muscle tissue itself was simplified as square columns in cross-section, with the muscle bundle membranes tightly enclosing the muscle bundles. This geometric representation of the mesoscopic muscle bundle–connective tissue complex is shown in Fig. 6.

Since only a localized region within the muscle tissue was analyzed for inter-scale parameter evaluation, a small portion of the muscle tissue in a non-weight-bearing sitting posture was extracted. The mesoscopic muscle model was idealized as a square with a cross-sectional side length of 4 mm, containing 25 muscle bundles.

The cylindrical muscle bundles were referred to by Marcucci *et al*. who measured the lateral femoral muscle geometry in older adults (Marcucci *et al.* 2019). Their study compared muscle bundle cross-sectional areas captured via imaging with those obtained from cryosections. For muscle bundles composed of three to six muscle fibers, the study reported an approximate circular cross-sectional radius of 0.07 mm. In this study, a microscopic-scale muscle bundle model comprising 37 muscle fibers was used, with a converted estimated radius of 0.3 mm. To ensure computational efficiency and consistency, the thickness of the mesoscopic muscle bundles was set to 4 mm, matching that of the macroscopic model.

#### Mechanical parameters

In this study, the Yeoh hyperelastic parameters and the Prony Series fourth-order viscoelastic parameters for connective tissues (CT) were obtained through a literature review. For muscle bundles, the Ogden hyperelastic parameters and the Prony Series second-order viscoelastic parameters were determined by fitting hyperelastic and viscoelastic models to the stress-strain and stress relaxation curves, respectively. These curves were derived from simulations of muscle bundle complexes, following the validation of hyperelastic and viscoelastic parameters of micro-modeled tissues based on literature data.

The hyperelastic and viscoelastic parameters of muscle bundles and connective tissues were assigned to the mesoscopic model (Then *et al.* 2012; Rahemi *et al.* 2014), respectively. Table 3 presented the elastic mechanical parameters for different regions at the macroscopic scale used in this study, while Table 4 provided the viscoelastic mechanical parameters for selected tissues at the macroscopic scale.

**Table 3:** Hyperelastic mechanical parameters of different regions of mesoscopic model

Region	Type	Constitutive model	ABAQUS parameter (Rahemi *et al.* 2014)
CT	Hyperelastic	Yeoh	$ c10=6.75\;\mathrm{k}\mathrm{P}\mathrm{a};c20=27.8\;{\mathrm{kPa}}; $ $ c30=-1.97\;\mathrm{k}\mathrm{P}\mathrm{a};D1=2.983 $
Bundle	Hyperelastic	Ogden	$ \mu 1=36\;\mathrm{k}\mathrm{P}\mathrm{a};\alpha 1=7.34;D1=1.12 $

**Table 4:** Viscoelastic mechanical parameters in different regions of mesoscopic model

Region	Type	Constitutive model	ABAQUS parameter (Then *et al.* 2012)
CT	Viscoelastic	Prony series	$ g1=7.67e-1;k1=1.17e-2;\tau 1=2 $ $ g2=6.44e-2;k2=2.29e-4;\tau 2=4e+1 $ $ g3=6.08e-4;k3=3.74e-4;\tau 3=8e+1 $ $ g4=2.18e-2;k4=1.29e-5;\tau 4=2e+2 $
Bundle	Viscoelastic	Prony series	$ g1=2.05e-1;k1=0;\tau 1=0.13 $ $ g2=2.02e-1;k2=0;\tau 2=2.14 $

#### Mesh

To assess the meshing sensitivity of the mesoscopic model, one end was fixed while a strain of 5% of its length was applied to the other end. The resulting combined force exerted on the entire model was then evaluated.

Initially, with a mesh consisting of 17,868 elements for connective tissue and 918 elements for muscle bundles, the total applied force was calculated as 0.0495 N. When the mesh density was doubled – resulting in 35,267 elements for connective tissue while maintaining 918 cells for muscle bundles – the total applied force was 0.0498 N. This yielded an error of 0.74%.

Since the variation in mesh density resulted in an error of less than 1%, the selected meshing parameters for connective tissue and muscle bundles were deemed appropriate for the study.

### Microscopic-scale muscle fibers and extracellular matrix complex

#### Geometric modeling

The endomysium surrounding muscle fibers forms a highly ordered polygonal lattice structure, which allows for an efficient and compact arrangement of fibers within muscle bundles. To accurately represent this structure, muscle fibers were abstracted as hexagonal prisms in the microscopic model, ensuring a close-packed alignment within the bundles.

In the mesoscopic model, muscle bundles were modeled as cylindrical structures with a cross-sectional radius of 0.3 mm. For the microscopic model, the length of individual muscle bundles was set to 1.2 mm to accommodate the scale of microscopic modeling. The resulting geometric model of the microscopic-scale muscle fiber and extracellular matrix complex is illustrated in Fig. 6.

Given the physiological dimensions of muscle fibers, which typically range from 50 to 80 μm in diameter (Röhrle *et al.* 2019), this study set the orthogonal diagonal of the hexagonal prism cross-section to 70 μm. A total of 37 muscle fibers were incorporated into the muscle bundle model to accurately reflect muscle bundle composition.

#### Mechanical parameters

Both extracellular matrix (ECM) and muscle fibers exhibit hyperelastic and viscoelastic behaviors, requiring a combined approach for accurate mechanical characterization. In this study, the Yeoh hyperelastic model was selected to describe the hyperelastic properties of the ECM, while the Ogden hyperelastic model was used for muscle fibers. For viscoelasticity, the Prony series viscoelastic model was applied to both the ECM and muscle fibers.

The hyperelastic parameters of the Ogden model and the third-order Prony series viscoelastic parameters for both ECM and muscle fibers were obtained through a literature review. Additionally, the consistency of the mechanical behavior across microscopic, mesoscopic, and macroscopic models was verified by ensuring that the parameters derived from the microscopic model were in agreement with those used in the larger-scale models.

The hyperelastic and viscoelastic parameters for the extracellular matrix and muscle fibers were assigned to the microscopic model (Zhang *et al.* 2020; Lim *et al.* 2019; Fallah *et al.* 2017). Table 5 presented the hyperelastic mechanical parameters for different regions at the macroscopic scale, while Table 6 provided the viscoelastic mechanical parameters for selected tissues at the macroscopic scale.

**Table 5:** Hyperelastic mechanical parameters of different regions of the microscopic model

Region	Type	Constitutive model	ABAQUS parameter
ECM	Hyperelastic	Yeoh	$ C10=28.4 \;{\mathrm{kPa}};C20=0.13 \;{\mathrm{Mpa}}; $ $ C30=76 \;{\mathrm{kPa}};D1=0.709 $
Fiber	Hyperelastic	Ogden	$ \mu 1=2.9\;\mathrm{k}\mathrm{P}\mathrm{a};\alpha 1=5.11;D1=13.48 $

**Table 6:** Viscoelastic mechanical parameters in different regions of the microscopic model

Region	Type	Constitutive model	ABAQUS parameter (Fallah *et al.* 2017)
ECM	Viscoelastic	Prony series	$ g1=0.24;k1=0;\tau 1=0.1 $ $ g2=0.16;k2=0;\tau 2=1 $ $ g3=0.11;k3=0;\tau 3=10 $
Fiber	Viscoelastic	Prony series	$ g1=0.19;k1=0;\tau 1=0.1 $ $ g2=0.13;k2=0;\tau 2=1 $ $ g3=0.1;k3=0;\tau 3=10 $

#### Mesh

To verify the mesh sensitivity of the microscopic model, one end of the model was fixed while a 5% strain was applied to the other end. The resulting combined force on the entire model was analyzed. Initially, the ECM and muscle fibers were meshed with 28,766 and 374 elements, respectively, yielding a combined force of 0.000784 N. When the mesh density was doubled (ECM: 73050 elements, muscle fibers: 374 elements), the computed force was 0.000783 N, with an error of only 0.11%. Since the variation in force remained below 1% across different mesh densities, the chosen meshing strategy for the ECM and muscle fibers was deemed reasonable.

### Derivation of displacement loading across different scales

The simulation was conducted in ABAQUS FE software. Essentially, stress and strain transfer across scales is the transmission of deformation. Therefore, to capture the evolution of the mesoscopic model under a macroscopic static load, the displacement for the mesoscopic model must be derived from the macroscopic model. And the displacement for the microscopic model must be derived from the mesoscopic model. A square region was selected around the node within the macroscopic muscle model that exhibits the maximum Von Mises stress. The displacement of the surrounding area is then calculated. Since the cross-sectional properties are consistent across different thicknesses, it is sufficient to compute the displacements at the four vertices of a single cross-section via the deformation process. The average displacements of the top and bottom surfaces of the mesoscopic model are then computed to obtain the relative displacement induced by the deformation process. The displacements at the four nodes are analyzed individually, so the average compression ratio of these edges was then used to determine the mesoscopic model’s displacement load. The displacement load on the microscopic model was calculated with the same approach.

### Cyclic force paradigm

The cyclic compressive force stimulus was applied to the cushion at the macroscopic scale. In practice, this was implemented as a displacement vibration setup on the cushion, essentially translating the cyclic mechanical stimulus into cyclic displacement loading.

For cyclic force frequency, several values were previously applied to viscoelastic muscle cells, as reported by Jia (Jia *et al.* 2022), who used frequencies of 0.02, 0.1, 0.25, 0.5, 0.75, 1, 5 and 10 Hz. In the present study, 0.25 and 1 Hz were selected as low-frequency values for cyclic displacement loading. Additionally, Wong *et al*. applied intermittent vibration at 35 Hz to protect aging muscle tissue from mechanical and oxidative damage, and this study adopted 30 Hz as the high-frequency value for cyclic displacement loading (Wong *et al.* 2017).

For cyclic force amplitude, three values were chosen: 0.5, 1, and 2 mm. The vibration was applied after the initial analysis step (0.1 s) and compared with a static stress-relaxation curve (without cyclic displacement loading) to examine the effects of periodic vibration frequency and amplitude on the average maximal Von Mises stresses at the macro-, meso-, and microscale levels, as well as the average minimum principal stresses at the microscale.

**Figure 7:**
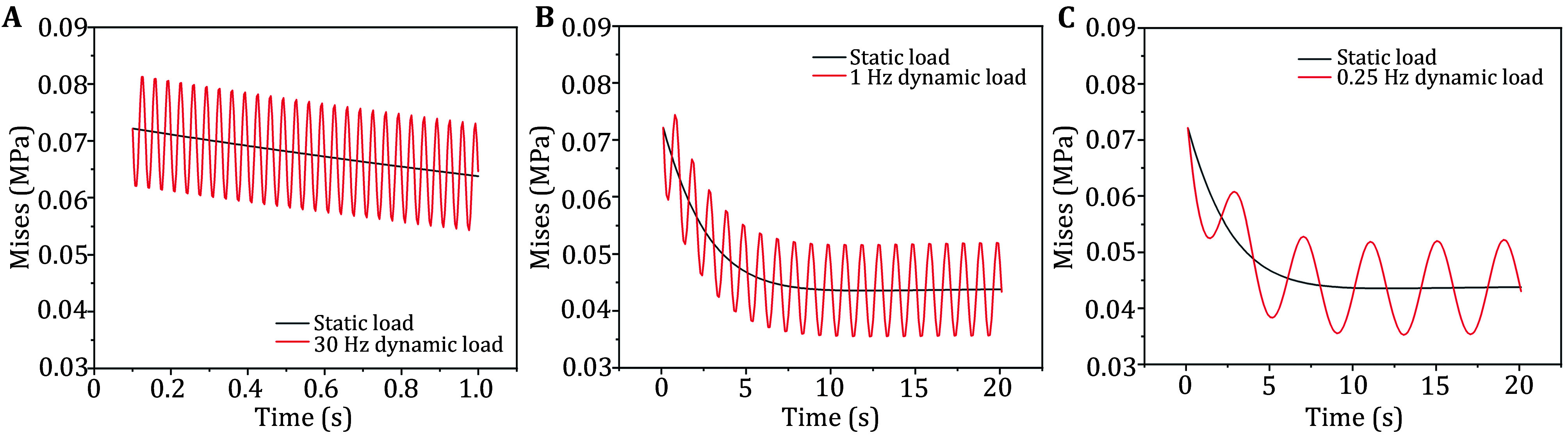
Von Mises stress changing over time under static and dynamic loading conditions at 30 Hz (**A**), 1 Hz (**B**), and 0.25 Hz (**C**) in macroscopic model

Due to slight deformation of the upper surface of the cushion caused by the downward displacement loading of the sciatic tuberosity, periodic displacements were applied to the lower surface of the cushion, where higher stiffness was considered. The vibration was implemented as a sinusoidal function with cyclic force paradigms at 0.25, 1 and 30 Hz, each having amplitude values of 0.5, 1 and 2 mm. Fig. 7 indicated Von Mises stress changing over time under static and dynamic loading conditions at 0.25, 1 and 30 Hz in the macroscopic model.

## Conflict of interest

Xue Ke, Yansong Lu, Linjing Peng, Lu Yu and Yifei Yao declare that they have no conflict of interest.

## References

[bBouten2005] Bouten C, Oomens C, Colin D, Bader D (2005) The aetiopathology of pressure ulcers: a hierarchical approach. In: Bader DL, Bouten CVC, Colin D, Oomens CWJ (eds. ) Pressure ulcer research: current and future perspectives. Berlin, Heidelberg: Springer, pp 1–9

[bBouten2003] Bouten CV, Oomens CW, Baaijens FP, Bader DL (2003) The etiology of pressure ulcers: skin deep or muscle bound? Arch Phys Med Rehabil 84(4): 616–619

[bBrandeis1990] (1990). The epidemiology and natural history of pressure ulcers in elderly nursing home residents. JAMA.

[bBreuls2003] (2003). Compression induced cell damage in engineered muscle tissue: an *in vitro* model to study pressure ulcer aetiology. Ann Biomed Eng.

[bBursa2006] (2006). FE models of stress-strain states in vascular smooth muscle cell. Technol Health Care.

[bCen2021] (2021). Multiscale mechanical responses of young and elderly human femurs: a finite element investigation. Bone.

[bColeman2014] (2014). A new pressure ulcer conceptual framework. J Adv Nurs.

[bDaniel1981] (1981). Etiologic factors in pressure sores: an experimental model. Arch Phys Med Rehabil.

[bDugan2014] Dugan JM, Cartmell SH, Gough JE (2014) Uniaxial cyclic strain of human adipose–derived mesenchymal stem cells and C2C12 myoblasts in coculture. J Tissue Eng 5: 2041731414530138. https://doi.org/10.1177/2041731414530138

[bFallah2017] (2017). Rate-dependent behavior of connective tissue through a micromechanics-based hyper viscoelastic model. Int J Eng Sci.

[bHaalboom2005] Haalboom J (2005) Medical perspectives in the 21st century. In: Bader DL, Bouten CVC, Colin D, Oomens CWJ (eds.) Pressure ulcer research: current and future perspectives. Berlin, Heidelberg: Springer, pp 11–21

[bIchioka2011] (2011). *In vivo* analysis of skin microcirculation and the role of nitric oxide during vibration. Ostomy Wound Manage.

[bJia2022] (2022). Cell membrane tensile strain under cyclic compression: a viscoelastic myoblast finite element model. Med Novel Technol Devices.

[bJia2023] (2023). Stiffening of the gluteal muscle increased the intramuscular stress: an in-silico implication of deep tissue injury. Heliyon.

[bJiang2014] (2014). The incidence, risk factors and characteristics of pressure ulcers in hospitalized patients in China. Int J Clin Exp Pathol.

[bLi2009] (2009). Cyclic force upregulates mechano-growth factor and elevates cell proliferation in 3D cultured skeletal myoblasts. Arch Biochem Biophys.

[bLim2019] (2019). Passive force and viscoelastic properties of single fibers in human aging muscles. Eur J Appl Physiol.

[bLinderGanz2007] (2007). Assessment of mechanical conditions in sub-dermal tissues during sitting: a combined experimental-MRI and finite element approach. J Biomech.

[bMak2011] (2011). Deformation and reperfusion damages and their accumulation in subcutaneous tissues during loading and unloading: a theoretical modeling of deep tissue injuries. J Theor Biol.

[bMarcucci2019] (2019). Fibre and extracellular matrix contributions to passive forces in human skeletal muscles: an experimental based constitutive law for numerical modelling of the passive element in the classical Hill-type three element model. PLoS One.

[bNola1980] (1980). Differential response of skin and muscle in the experimental production of pressure sores. Plast Reconstr Surg.

[bPeeters2005] (2005). Viscoelastic properties of single attached cells under compression. J Biomech Eng.

[bRahemi2014] (2014). Regionalizing muscle activity causes changes to the magnitude and direction of the force from whole muscles—a modeling study. Front Physiol.

[b24] Reese SP, Weiss JA (2015) Tendons and ligaments: current state and future directions. In: De S, Hwang W, Kuhl E (eds.) Multiscale modeling in biomechanics and mechanobiology. London: Springer, pp 159−206

[bRhrle2019] (2019). Multiscale modeling of the neuromuscular system: coupling neurophysiology and skeletal muscle mechanics. WIREs Syst Biol Med.

[bSari2015] (2015). Vibration inhibits deterioration in rat deep‐tissue injury through HIF 1–MMP axis. Wound Repair Regener.

[bSoltow2010] (2010). Nitric oxide regulates stretch-induced proliferation in C2C12 myoblasts. J Muscle Res Cell Motil.

[bTakemoto2012] (2012). The number of cyclic stretch regulates cellular elasticity in C2C12 myoblasts. CellBio.

[bThen2012] (2012). Method for characterizing viscoelasticity of human gluteal tissue. J Biomech.

[bTubaishat2018] (2018). Pressure ulcers prevalence in the acute care setting: a systematic review, 2000–2015. Clin Nurs Res.

[bWang2004] (2004). Fibroblast responses to cyclic mechanical stretching depend on cell orientation to the stretching direction. J Biomech.

[bWang2015] (2015). Strain amplification analysis of an osteocyte under static and cyclic loading: a finite element study. BioMed Res Int.

[bWang2016] (2016). Dynamic analyses of osteoblast vibrational responses: a finite element viscoelastic model. J Vibroeng.

[bWang2019] (2019). CTGF regulates cyclic stretch-induced vascular smooth muscle cell proliferation via microRNA-19b-3p. Exp Cell Res.

[bWang2010] (2010). Up-regulated α-actin expression is associated with cell adhesion ability in 3-D cultured myocytes subjected to mechanical stimulation. Mol Cell Biochem.

[bWei2021] (2021). The prevalence and prevention of pressure ulcers: a multicenter study of nine nursing homes in eastern China. J Tissue Viability.

[bWong2017] (2017). Intermittent vibration protects aged muscle from mechanical and oxidative damage under prolonged compression. J Biomech.

[bXiao2014] (2014). Accumulation of loading damage and unloading reperfusion injury—modeling of the propagation of deep tissue ulcers. J Biomech.

[bYao2016] (2016). Strengthening of C2C12 mouse myoblasts against compression damage by mild cyclic compressive stimulation. J Biomech.

[bYao2015] (2015). The effects of oxidative stress on the compressive damage thresholds of C2C12 mouse myoblasts: implications for deep tissue injury. Ann. Biomed. Eng..

[bZhang2007] (2007). Effect of cyclic stretch on β_1D_-integrin expression and activation of FAK and RhoA. Am J Physiol-Cell Physiol.

[bZhang2020] (2020). Microstructural analysis of skeletal muscle force generation during aging. Int J Numer Methods Biomed Eng.

[bZhu2000] (2000). Cell mechanics: mechanical response, cell adhesion, and molecular deformation. Ann Rev Biomed Eng.

